# HER2-targeted dual radiotracer approach with clinical potential for noninvasive imaging of trastuzumab-resistance caused by epitope masking

**DOI:** 10.7150/thno.74154

**Published:** 2022-07-18

**Authors:** Liqiang Li, Tianyu Liu, Linqing Shi, Xin Zhang, Xiaoyi Guo, Biao Hu, Meinan Yao, Hua Zhu, Zhi Yang, Bing Jia, Fan Wang

**Affiliations:** 1 Medical Isotopes Research Center and Department of Radiation Medicine, School of Basic Medical Sciences, Peking University, Beijing, 100191, China.; 2 Key Laboratory of Carcinogenesis and Translational Research (Ministry of Education/Beijing), Department of Nuclear Medicine, Peking University Cancer Hospital & Institute, Beijing 100142, China.; 3 Department of Integration of Chinese and Western Medicine, School of Basic Medical Sciences, Peking University, Beijing, 100191, China.; 4 Key Laboratory of Protein and Peptide Pharmaceuticals, CAS Center for Excellence in Biomacromolecules, Institute of Biophysics, Chinese Academy of Sciences, Beijing 100101, China.; 5 Bioland Laboratory (Guangzhou Regenerative Medicine and Health Guangdong Laboratory), Guangzhou 510005, China.

**Keywords:** SPECT/CT imaging, VHH, Trastuzumab, HER2-targeted therapy

## Abstract

**Rationale:** The decreased HER2-accessibility by epitope masking is a primary trastuzumab-resistance mechanism. In this study, we developed a HER2-targeted dual radiotracer approach to predict the HER2-trastuzumab engagement noninvasively.

**Methods:** Two novel HER2-specific VHHs, MIRC208 and MIRC213, were acquired by immunizing alpaca with human HER2 protein, and were site-specifically labeled with ^99m^Tc. Biodistribution and SPECT/CT imaging studies were performed in mice bearing HER2-positive and HER2-negative tumors. The HER2 binding sites of ^99m^Tc-MIRC208 and ^99m^Tc-MIRC213 were investigated by cell binding and SPECT/CT imaging studies. We evaluated the therapeutic predictive ability of our dual-radiotracer imaging approach for trastuzumab treatment in mice bearing MUC4-positive tumors (trastuzumab-resistant JIMT-1 and 87MUC4) and MUC4-negative tumors (trastuzumab-sensitive 7HER2 and NCI-N87). The preliminary clinical studies of ^99m^Tc-MIRC208 were performed in two patients with HER2-positive breast tumors.

**Results:** ^99m^Tc-MIRC208 and ^99m^Tc-MIRC213 clearly visualized HER2-positive tumors, but not HER2-negative tumors. ^99m^Tc-MIRC208 competes with trastuzumab for HER2-binding while ^99m^Tc-MIRC213 recognizes HER2 on an epitope that is not masked by MUC4. The SPECT/CT studies with ^99m^Tc-MIRC208 and ^99m^Tc-MIRC213 clearly showed that the MUC4-negative and trastuzumab-sensitive 7HER2 and NCI-N87 tumors had very similar tumor uptake with the SUV_208_/SUV_213_ (2 h) ratios of 1.11 ± 0.17 in 7HER2 and 1.25 ± 0.22 in NCI-N87. However, the MUC4-positive JIMT-1 tumors showed the decreased SUV_208_/SUV_213_ (2 h) ratio (0.63 ± 0.07), which correlated well with the low response rate to trastuzumab therapy. The SUV_208_/SUV_213_ (2 h) ratio was reduced to 0.72 ± 0.02 in MUC4-expressing NCI-N87 cells, and resulting in the decreased trastuzumab sensitivity, further supporting the correlation between the SUV_208_/SUV_213_ (2 h) ratio and trastuzumab-sensitivity. The primary and metastatic HER2-positive lesions of patients were clearly visualized by ^99m^Tc-MIRC208 SPECT at 2 h post injection.

**Conclusion:** Overall, we demonstrated that the dual radiotracer imaging strategy is a valid noninvasive approach for the cancer patient selection before trastuzumab therapy. ^99m^Tc-MIRC213 SPECT is utilized to quantify the tumor HER2 expression and screen HER2-positive cancer patients, while ^99m^Tc-MIRC208 SPECT is used to determine the HER2-accessibility of trastuzumab. The SUV_208_/SUV_213_ (2 h) ratio is an important biomarker to determine the responsiveness of trastuzumab therapy.

## Introduction

Human epidermal growth factor receptor 2 (HER2) is an important therapeutic target for many types of cancers 1, 2. Targeted therapy with trastuzumab (Traz), a humanized IgG monoclonal antibody against the HER2 extracellular domain (ECD), has become a mainstay for patients with HER2-positive breast cancer (BC) and gastric cancer (GC) 3, 4. However, only a small fraction of cancer patients is responsive to the Traz therapy due to the inherent and/or acquired resistance mechanisms 1, 2, 4-6. One of the resistance mechanisms is the decreased binding of Traz to HER2 caused by epitope masking 1, 7-11. It was reported that the membrane-associated mucin 4 (MUC4) can dimerize with HER2 and sterically impede the Traz-binding of its epitope on HER2 receptors, leading to significant resistance to Traz therapy 8, 9, 12. In a recent study, it was found that MUC4 overexpression in tumors is strongly correlated with shorter disease-free survival (DFS) in the HER2-positive BC patients receiving adjuvant Traz treatment 7. Therefore, determination of the HER2-accessibility is essential for improvement of the response rate prior to Traz treatment 2.

In clinical practice, HER2 status is generally determined by immunohistochemistry (IHC) and fluorescence in situ hybridization (FISH). However, IHC and FISH provide no information with regard to HER2-accessibility because the antibodies used in IHC procedures target HER2 intracellular domain (ICD), and FISH could only detect the HER2 gene amplification status 13-17. Positron emission tomography (PET) with the radiolabeled Traz is capable of mapping the HER2 expression and directly reflecting HER2-accessibility of trastuzumab 18-23. However, the poor pharmacokinetic characteristics (e.g. slow clearance from blood circulation and long retention times in non-target tissues) of the antibody-based radiotracers severely limit their widespread clinical utility 24-27.

Single-domain variable region (VHH) is the smallest antibody fragment derived from camelid heavy chain-only antibodies. The VHH possesses many unique features (e.g. good antigen affinity, fast renal clearance and high tissue penetration), which make it better suited than the full-length antibody as the targeting moiety for radiotracer development 28, 29. In this study, we generated two HER2-specific radiotracers ^99m^Tc-MIRC213 and ^99m^Tc-MIRC208. ^99m^Tc-MIRC213 was a Traz-noncompetitor useful for selection of the HER2-positive patients and quantify the tumor HER2 expression. ^99m^Tc-MIRC208 is a Traz-competitor useful for the determination of HER2-accessibility of Traz because its epitope on HER2 is masked by MUC4. The combination of ^99m^Tc-MIRC213 and ^99m^Tc-MIRC208 could be a powerful noninvasive tool to select responders to Traz therapy before treatment initiation.

## Results

### Generation of novel HER2-specific ^99m^Tc radiotracers

**Figure 1:**
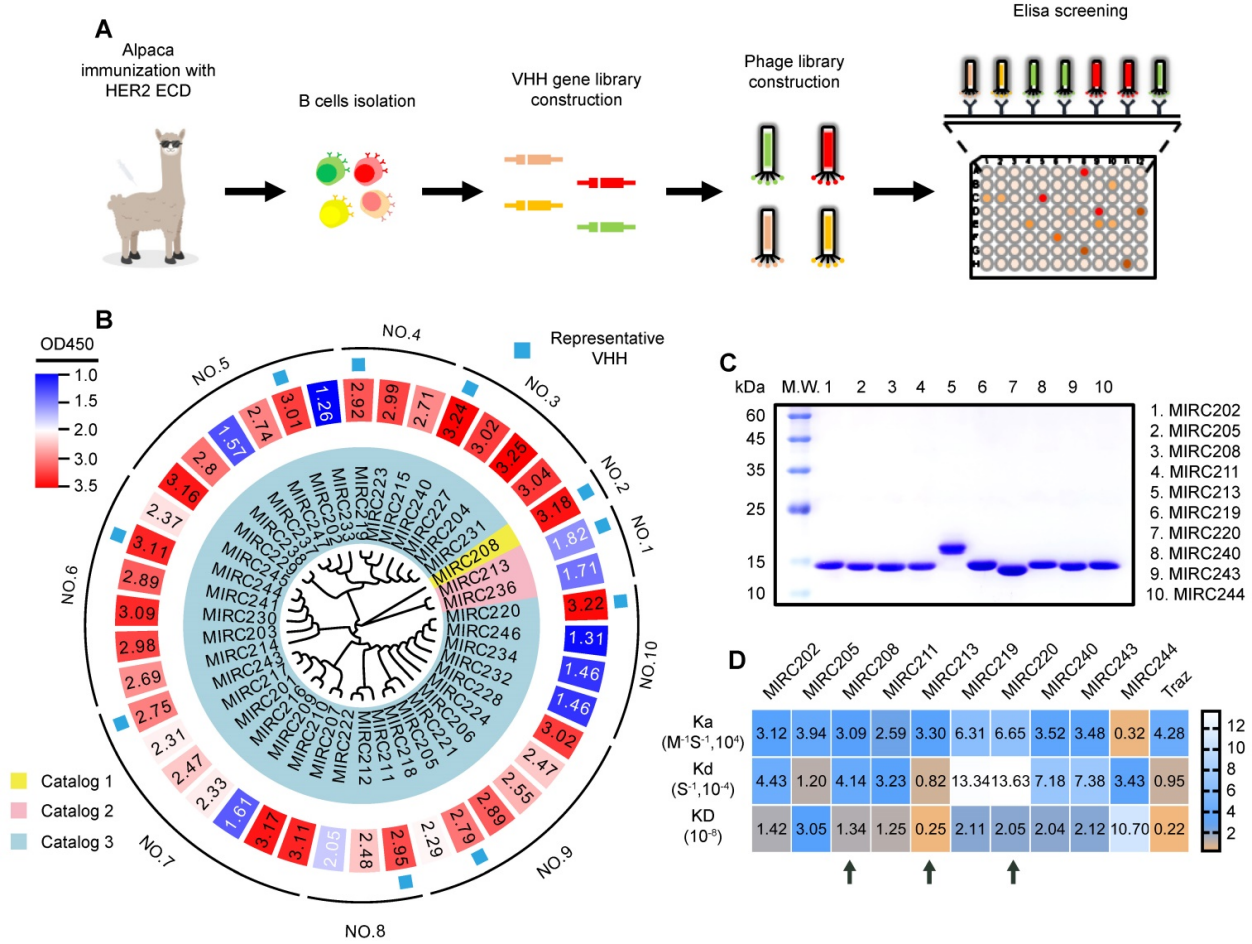
** Immunization of alpaca with recombinant human HER2 ECD protein yields potent HER2-recognized VHHs. (A)** Schematic representation of HER2-specific VHH generation. **(B)** Phylogenetic tree of VHHs based on the sequence homology analysis, and the heatmap of HER2 affinities of 42 VHHs determined by phage display. Forty-two VHHs against to HER2 ECD were acquired and can be divided into 10 groups or 3 catalogs according to the whole sequence homology or CDR3 sequence homology, respectively. **(C)** The purities of 10 representative VHHs were analyzed by SDS-PAGE study and were greater than 90%. **(D)** SPRi experiment for the measurement of HER2 binding affinities of 10 representative VHHs. The kinetic association constant (Ka), dissociation constant (Kd) and KD are shown as heatmaps. Traz is the positive control. Three lead VHHs, MIRC208, MIRC213 and MIRC220, are indicated by black arrows.

A library containing 42 individual VHHs were generated from an alpaca immunized with human HER2 ECD protein (Figure 1A). These VHHs could be divided into 10 groups based on the full-length sequence homology. We randomly selected one VHH from each group for production and purification in an *E. coli* expression system (Figure 1B). The purity of 10 selected VHHs were verified by SDS-PAGE (Figure 1C). The surface plasmon resonance (SPR) experiment was performed to evaluate the binding affinity of 10 selected VHHs against the recombined human HER2 ECD protein. Traz and a VHH against other receptor were chosen as the positive and negative controls, respectively, to validate the method (Figure 1D and Figure S1). We found that all 10 VHHs showed excellent binding affinity for HER2. MIRC244 had a relatively low affinity for HER2. MIRC213 has the best HER2-binding affinity with Kd value at the 10^-9^ M level. Other VHHs bind to HER2 protein with the HER2-affinity in 10^-8^ M range. Since VHHs with different sequences of complementarity determining region 3 (CDR3) are likely to recognize different target epitopes, these VHHs were further divided into 3 classes on the basis of the alignment of the CDR3 sequence. MIRC208, MIRC213 and MIRC220 were selected as the lead compounds for subsequent studies (Figure 1C-D).

**Figure 2:**
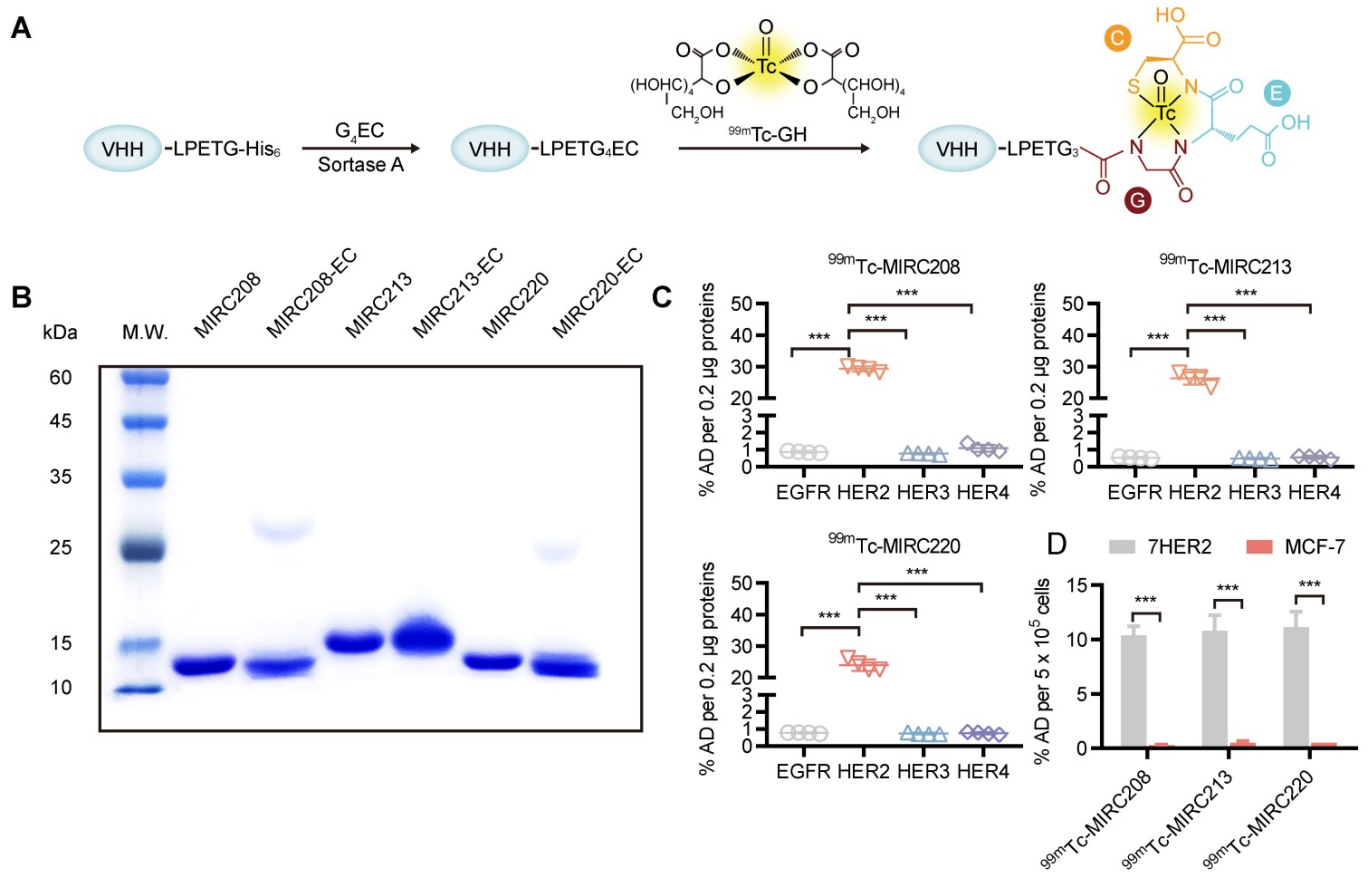
** Site-specific ^99m^Tc-labeling of VHHs that specifically bind to the human HER2 protein and HER2-overexpressing cancer cells. (A)** VHHs were labeled with ^99m^Tc by Sortase A-mediated transpeptidation. **(B)** A protein gel of characterization of MIRC208-EC, MIRC213-EC and MIRC220-EC. **(C)** and **(D)** Binding of three ^99m^Tc-VHHs to recombinant human HER family ECD proteins **(C)** and HER2-high or HER2-low BC cell lines **(D)**. 7HER2 is a HER2-overepxressing BC cell line acquired by stable HER2 gene transfection in MCF-7 cells. All ^99m^Tc-VHHs only bind to human HER2 ECD with avidity, and the affinities of all ^99m^Tc-VHHs are significantly greater for 7HER2 than for MCF-7 (n = 4). The error bars represent standard deviation. ***p < 0.001, student's paired *t test*. %AD, % added radioactive dose.

MIRC208, MIRC213 and MIRC220 were genetically engineered to contain a C-terminus Sortase A recognition tag, LPETG. The G_4_EC oligopeptide was conjugated to the C-terminus of the modified VHHs using Sortase A-mediated transacylation to generate the expected VHH-ECs bioconjugates for ^99m^Tc-radiolabeling (Figure 2A). Successful bioconjugation was verified by LC-MS analysis (Figure S2). The purity of VHH-ECs was confirmed by SDS-PAGE (Figure 2B). The ^99m^Tc-labeling was achieved by reacting VHH-EC bioconjugate with the ^99m^Tc-GH (GH = glucoheptate) intermediate at 37 °C for 0.5 h to yield the corresponding ^99m^Tc radiotracers ^99m^Tc-MIRC208, ^99m^Tc-MIRC213 and ^99m^Tc-MIRC220 (Figure 2A). Their radiochemical purity prior to purification was 89.23 ± 2.58%, 90.12 ± 1.22% and 89.92 ± 2.91%, respectively (Figure S3A). All three radiotracers were stable for >6 h in saline at 25 °C and retained their integrity in mice blood at 6 h post injection (Figure S3B). The *in vitro* binding assays were performed to test their HER2 affinity (Figure 2C). The results clearly showed that ^99m^Tc-MIRC208, ^99m^Tc-MIRC213 and ^99m^Tc-MIRC220 are all able to bind specifically to HER2 ECD and the HER2-expressing 7HER2 BC cells rather than the other receptors in the HER family, murine HER2 ECD and HER2-low MCF-7 cells (Figure 2C-D and Figure S4).

### Evaluation of ^99m^Tc radiotracers in tumor xenografts

**Figure 3:**
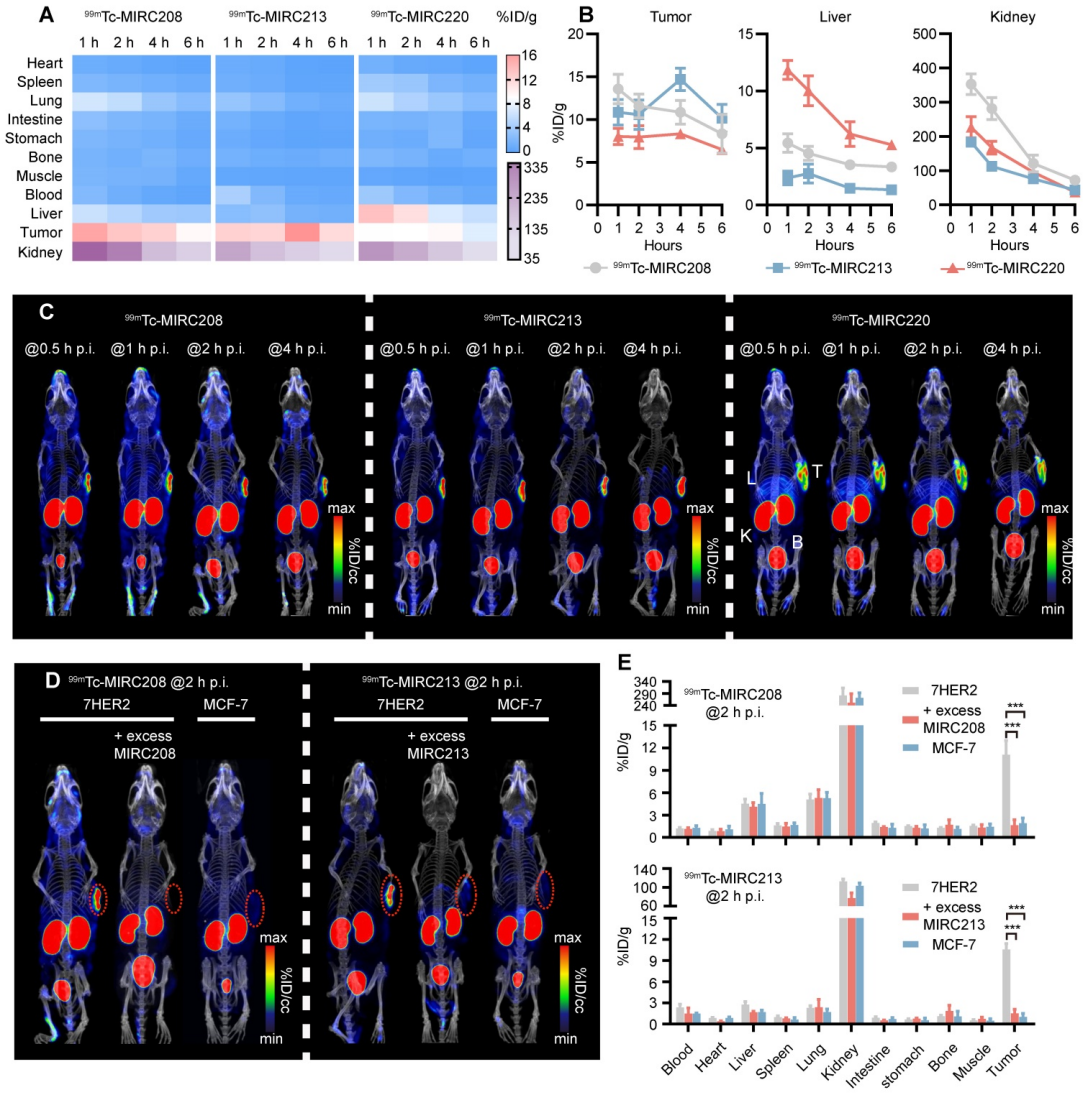
** All ^99m^Tc radiotracers selectively bind to 7HER2 tumors *in vivo* with different biodistribution properties. (A)** Heatmap of accumulation patterns of three ^99m^Tc-VHHs in 7HER2 tumor models at different time points. The color key represents the mean value of tracer accumulation in organs (%ID/g, n = 4 for each group). **(B)** The uptake differences of three ^99m^Tc-VHHs in the tumor, liver and kidney. **(C)** The noninvasive SPECT/CT imaging of ^99m^Tc-VHHs in 7HER2 tumor models at different time points. T, tumor; L, liver; K, kidney; B, bladder. SPECT/CT images **(D)** and biodistribution **(E)** of ^99m^Tc-MIRC208 and ^99m^Tc-MIRC213 in HER2-low MCF-7 and HER2-high 7HER2 xenografts with or without excess homologous VHH (n=4). The error bars represent standard deviation. ***p < 0.001, student's paired *t test*. Tumors were indicated by red circles.

^99m^Tc-MIRC208, ^99m^Tc-MIRC213 and ^99m^Tc-MIRC220 were evaluated for their capability to image HER2-positive tumors. Biodistribution studies were performed in 7HER2 tumor-bearing mice at multiple time points. It was found that all three ^99m^Tc radiotracers displayed a high tumor uptake (Figure 3A). The maximum uptake of ^99m^Tc-MIRC208 and ^99m^Tc-MIRC220 in the 7HER2 tumors was observed at 1 h p.i. (^99m^Tc-MIRC208: 13.58 ± 1.73 %ID/g, ^99m^Tc-MIRC220 8.03 ± 0.95 %ID/g). ^99m^Tc-MIRC213 showed a slow binding rate for 7HER2 tumors and reached maximum tumor accumulation at 4 h p.i. (14.69 ± 1.31 %ID/g). The difference in tumor binding rates between three ^99m^Tc-VHHs may be caused by their different KD values. ^99m^Tc-MIRC208 and ^99m^Tc-MIRC220 bound to 7HER2 tumors faster because they showed greater KD values than ^99m^Tc-MIRC213. There was a low uptake in normal organs (except liver and kidneys) with rapid clearance for all three ^99m^Tc radiotracers. At 1 h and 2 h p.i., ^99m^Tc-MIRC208 had the kidney uptake of 352.68 ± 30.31 and 281.47 ± 31.80 %ID/g, respectively, which were significantly higher than that of ^99m^Tc-MIRC213 and ^99m^Tc-MIRC220 (Figure 3B). ^99m^Tc-MIRC220 also showed lower tumor uptake and higher liver uptake at all the time points.

SPECT/CT studies were performed in the 7HER2 tumor-bearing model to illustrate the capability of ^99m^Tc-MIRC208, ^99m^Tc-MIRC213 and ^99m^Tc-MIRC220 as radiotracers to visualize HER2-positive tumors. It was found that all three ^99m^Tc radiotracers have significant uptake in the HER2-positive 7HER2 tumors (Figure 3C) as early as 0.5 h after injection, which is in complete agreement with the results from biodistribution. ^99m^Tc-MIRC208 has more kidney uptake than other two ^99m^Tc radiotracers. High liver and low tumor uptake of ^99m^Tc-MIRC220 was also observed at all the time points. Therefore,^ 99m^Tc-MIRC220 was eliminated from subsequent studies due to its unfavorable biodistribution.

SPECT/CT and biodistribution studies were also carried out in the HER2-negative MCF-7 tumor model to demonstrate the tumor specificity of ^99m^Tc-MIRC208 and ^99m^Tc-MIRC213 (Figure 3D-E). Blocking studies were performed using excess VHH in 7HER2 tumor model. At 2 h after injection, there was no radioactivity could be detected in the HER2-negative MCF-7 tumors and a close to 90% reduction in tumor uptakes was observed upon the homologous VHH blockade (Figure 3D-E).

### ^99m^Tc-MIRC208 and ^99m^Tc-MIRC213 bind to HER2 with different epitopes

**Figure 4:**
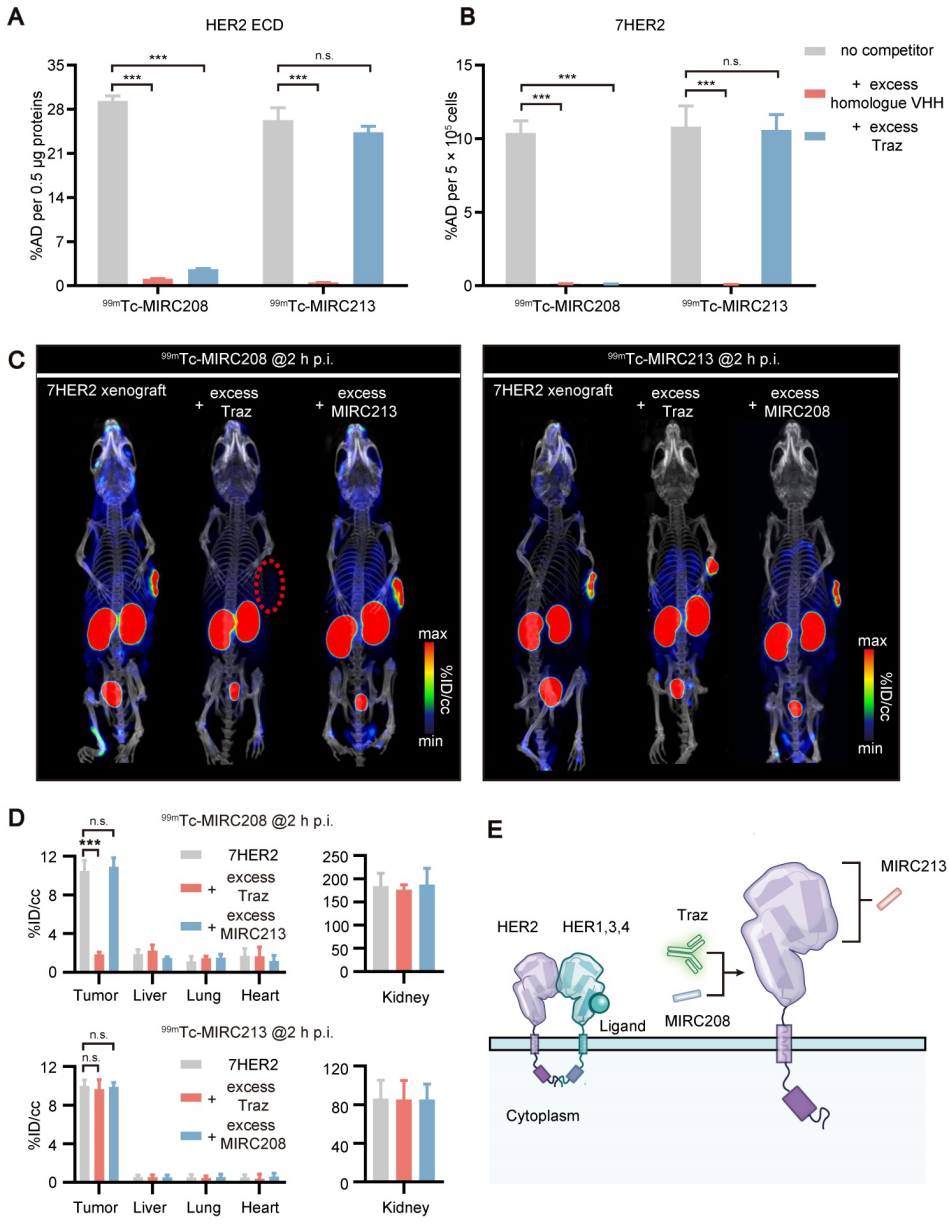
**^99m^Tc-MIRC208 and ^99m^Tc-MIRC213 bind to HER2 with different epitopes.** Binding of ^99m^Tc-MIRC208 and ^99m^Tc-MIRC213 to HER2 ECD protein **(A)** and 7HER2 **(B)** in the presence of excess homolog VHH or Traz (n=4). The error bars represent standard deviation. ***p < 0.001, student's paired *t test*. n.s., no significance. **(C)** SPECT/CT images of ^99m^Tc-MIRC208 and ^99m^Tc-MIRC213 in 7HER2 tumor-bearing mice treated with excess heterogeneous VHH (co-injection) or Traz (48 h earlier before radiotracer injection). **(D)** Biodistribution of SPECT signals in different organs from B. Values are expressed as %ID/cc (n=3). The error bars represent standard deviation. ***p < 0.001, student's paired *t test*. n.s., no significance. **(E)** Schematic representation of different HER2 binding epitopes of ^99m^Tc-MIRC208 and ^99m^Tc-MIRC213.

Human HER2 ECD consists of four subdomains I - IV. Traz recognizes the subdomain IV 3. Traz blocking studies were performed to explore the epitopes recognized by ^99m^Tc-MIRC208 and ^99m^Tc-MIRC213. It was found that Traz significantly inhibited the binding of ^99m^Tc-MIRC208 to the HER2 ECD protein (Figure 4A). In contrast, excess Traz had little effect on the uptake of^ 99m^Tc-MIRC213. The VHH blocking assays were used as a positive control and revealed the HER2 specificity of ^99m^Tc radiotracers. Next, we conducted the same binding assays in 7HER2 cells (Figure 4B). In agreement with the protein binding results, Traz significantly reduced the binding of ^99m^Tc-MIRC208 to 7HER2 cells, but not of ^99m^Tc-MIRC213. These results revealed the difference between ^99m^Tc-MIRC208 and ^99m^Tc-MIRC213 with respect to their HER2-binding epitopes. ^99m^Tc-MIRC208 may partially or fully bind to the subdomain IV of HER2 and compete with Traz while ^99m^Tc-MIRC213 binds to other subdomains (Figure 4E).

SPECT/CT studies were performed in the 7HER2 xenografts model to further confirm the results from* in vitro* studies. Traz was injected 48 h prior to co-injection of ^99m^Tc radiotracer and a homologous VHH blocker (Figure 4C-D). The SPECT/CT imaging studies were performed at 2 h p.i., because ^99m^Tc-MIRC208 and ^99m^Tc-MIRC213 showed similar uptakes in 7HER2 tumors at this time point (Figure 3A-B, ^99m^Tc-MIRC208: 11.58 ± 1.39 v.s. ^99m^Tc-MIRC213: 10.56 ± 1.72, *p* = 0.73). The HER2 binding of ^99m^Tc-MIRC208 was significantly inhibited by excess Traz and there was ~90% reduction in the 7HER2 tumor uptake. In contrast, the 7HER2 tumor uptake of ^99m^Tc-MIRC213 was not affected by excess Traz. ^99m^Tc-MIRC208 and ^99m^Tc-MIRC213 did not compete with each other in HER2 binding.

### Quantifying the tumor HER2 status and predicting resistance to Traz caused by MUC4 via a dual radiotracer approach

**Figure 5:**
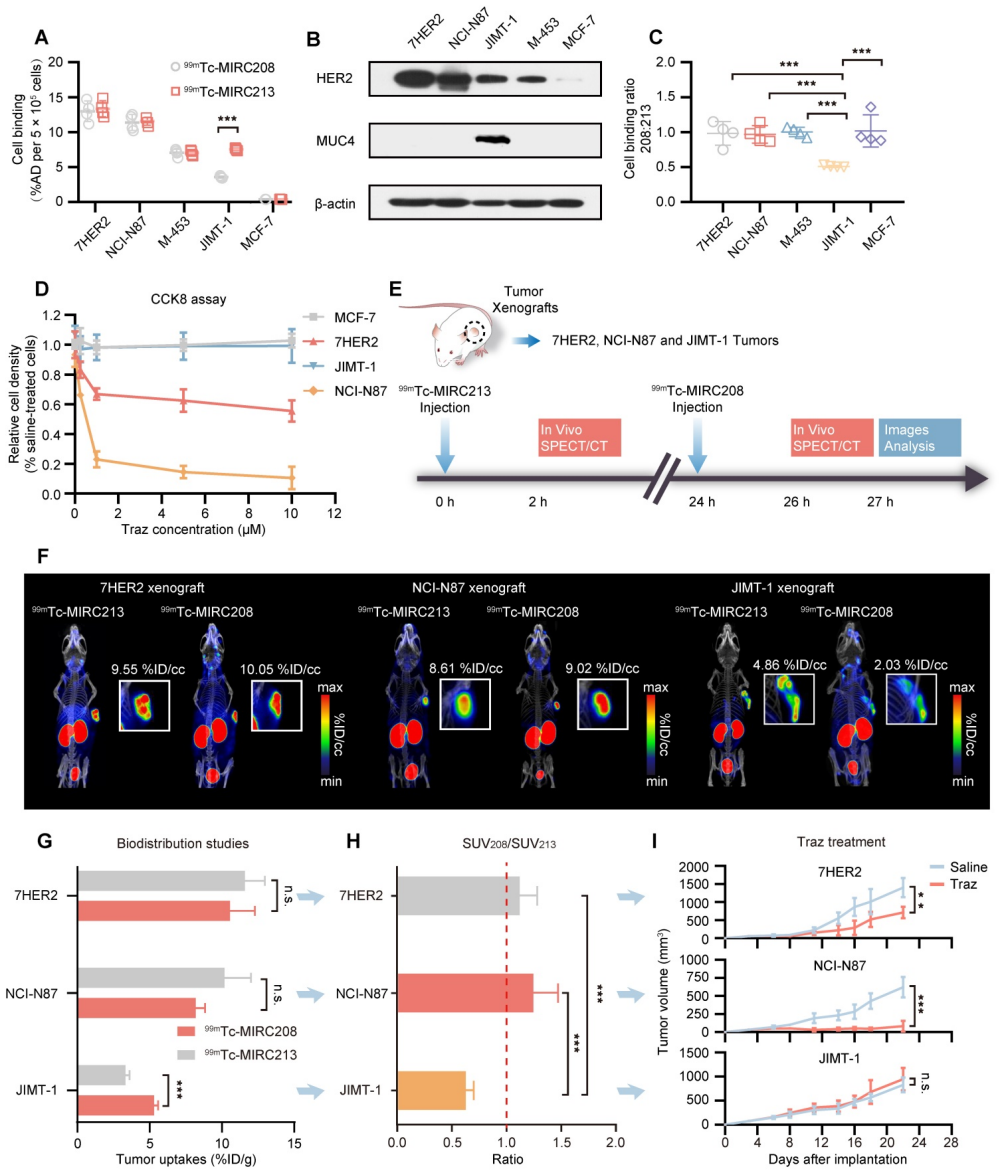
** HER2-Targeted dual radiotracer approach with ^99m^Tc-MIRC213 and ^99m^Tc-MIRC208 that could quantify HER2 status in tumors and select the responder to Traz prior to therapy. (A)** The uptakes of ^99m^Tc-MIRC208 and ^99m^Tc-MIRC213 in multiple BC and GC cell lines that express varying levels of HER2 receptor (n=4). The error bars represent standard deviation. ***p < 0.001, student's paired *t test*. **(B)** The levels of HER2 and MUC4 expression in various cell lines were analyzed by immunoblots. **(C)** The binding ratios of ^99m^Tc-MIRC208:^ 99m^Tc-MIRC213 in various cell lines. **(D)** *In vitro* therapeutic efficacy of Traz to different cell lines was determined by CCK-8 assay. Data represent the mean ± SD and were analyzed using two-way ANOVA coupled with a Bonferroni *post hoc* test; **p < 0.01, ***p < 0.001. **(E)** Schematic drawing illustrating the SPECT/CT imaging strategy with ^99m^Tc-MIRC213 and ^99m^Tc-MIRC208. The different uptake patterns of ^99m^Tc-MIRC208 and ^99m^Tc-MIRC213 in the same mice bearing 7HER2, NCI-N87 or JIMT-1 tumors were observed by *in vivo* SPECT/CT imaging **(F)** and *ex vivo* biodistribution **(G)**. **(H)** The different SUV_208_/SUV_213_ (2 h) ratios in 7HER2, NCI-N87 and JIMT-1 tumors. **(I)** Tumor growth curves of 7HER2, NCI-N87 and JIMT-1 xenografts treated with saline or Traz (n=7). Tumor volumes were expressed as the mean ± SD. **p < 0.01, ***p < 0.001, two-way analysis ANOVA followed by a Bonferroni* post hoc* test.

JIMT-1 is a well-known HER2-positive and MUC4-expressing BC cell line. The MUC4 receptor masks the epitope recognized by Traz 7-9, 30, 31. Therefore, JIMT-1-bearing animal models were utilized to explore the impact of MUC4 on the HER2-binding of ^99m^Tc-MIRC208 and ^99m^Tc-MIRC213. The results revealed that MUC4 could not prevent ^99m^Tc-MIRC213 from binding to HER2, and ^99m^Tc-MIRC213 could detect the HER2 expression in multiple cancer cell lines, which was completely consistent with the HER2 protein analysis (Figure 5A-B). The binding of ^99m^Tc-MIRC208 to JIMT-1 is much lower than that of ^99m^Tc-MIRC213, suggesting that MUC4 could mask the HER2-binding epitope of ^99m^Tc-MIRC208. Moreover, ^99m^Tc-MIRC208 and ^99m^Tc-MIRC213 accumulated similarly in the MUC4-negative cancer cells (Figure 5A-C). The results from the cytotoxicity assay revealed that MUC4-negative 7HER2 and NCI-N87 cells were sensitive to Traz treatment, and JIMT-1 cells showed resistance to Traz, which is similar to HER2-negative MCF-7 did (Figure 5D).

We designed an imaging approach that combined ^99m^Tc-MIRC213 and ^99m^Tc-MIRC208 (Figure 5E). SPECT/CT was performed using ^99m^Tc-MIRC213 in the tumor-bearing mice at 2 h post-injection, followed with ^99m^Tc-MIRC208 SPECT in the same animal at 24 h post-injection. These tumor-bearing mice had subcutaneous 7HER2 (sensitive), NCI-N87 (sensitive) and JIMT-1 (resistant) tumors, respectively. The uptake ratio of ^99m^Tc-MIRC208:^99m^Tc-MIRC213 in the same tumor was calculated by the regions of interests (ROI) analysis and defined as SUV_208_/SUV_213_ (2 h) value. ^99m^Tc-MIRC208 and ^99m^Tc-MIRC213 showed similar uptakes in the 7HER2 and NCI-N87 tumors. The SUV_208_/SUV_213_ (2 h) ratios in 7HER2 and NCI-N87 tumors were 1.05 ± 0.05 and 1.04 ± 0.11, respectively. However, there was a significant uptake decrease for ^99m^Tc-MIRC208 in the JIMT-1 tumor. The SUV_208_/SUV_213_ (2 h) in JIMT-1 was only 0.42 ± 0.02 (Figure 5F).

Biodistribution studies were performed in the same tumor-bearing models to further validate the results above. The uptake of ^99m^Tc-MIRC213 in 7HER2 and NCI-N87 tumors was 10.56 ± 1.72 and 8.19 ± 0.56 %ID/g, respectively. The uptake of ^99m^Tc-MIRC208 in 7HER2 and NCI-N87 tumors were 11.58 ± 1.39 %ID/g and 10.18 ± 1.83 %ID/g, respectively (Figure 5G and Figure S6). The SUV_208_/SUV_213_ (2 h) ratios in 7HER2 and NCI-N87 tumors were 1.11 ± 0.11 and 1.25 ± 0.22, respectively, and were significantly higher than 0.63 ± 0.07 in the JIMT-1 tumors (Figure 5H). The Traz treatment studies clearly showed that the 7HER2 and NCI-N87 tumors with high SUV_208_/SUV_213_ (2 h) ratios were sensitive to Traz therapy, while the JIMT-1 tumors with low SUV_208_/SUV_213_ (2 h) showed resistance to Traz treatment (Figure 5I).

**Figure 6:**
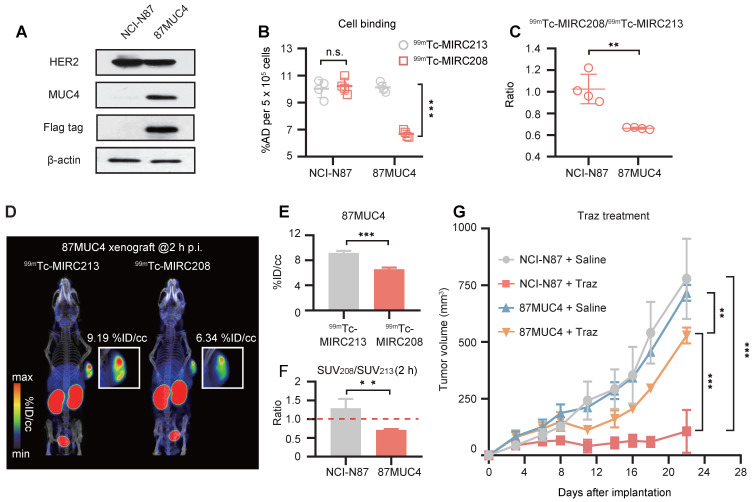
** Overexpression of MUC4 in NCI-N87 tumors decreases not only SUV_208_/SUV_213_ (2 h) but also the sensitivity to Traz treatment. (A)** MUC4 and HER2 levels of wild-type NCI-N87 and 87MUC4 cells were assessed in cell lysates by immunoblotting. **(B)** and **(C)** Both^ 99m^Tc-VHHs showed similar accumulation in wild-type NCI-N87 cells, but ^99m^Tc-MIRC213 bound to 87MUC4 at significantly higher levels than ^99m^Tc-MIRC208 (n = 4). The error bars represent standard deviation. ***p < 0.001, student's paired *t test*. **(D)** SPECT/CT imaging of ^99m^Tc-MIRC213 and ^99m^Tc-MIRC208 in the same 87MUC4 tumor bearing mice. **(E)** Quantification of SPECT signals of 87MUC4 tumors from C (n = 3). The error bars represent standard deviation. ***p < 0.001, student's paired *t test*. **(F)** SUV_208_/SUV_213_ (2 h) in wild-type NCI-N87 and 87MUC4 tumors (n = 3). error bars represent SD. **p < 0.01, student's paired *t test*. **(G)** Tumor growth curves of 87MUC4 and wild-type NCI-N87 xenografts treated with saline or Traz. Tumor volumes are expressed as the mean ± SD (n = 7). **p < 0.01, ***p < 0.001, two-way analysis ANOVA followed by a Bonferroni* post hoc* test.

It has been reported that the increased tumor MUC4 expression could inhibit the HER2-binding of Traz and reduce the Traz-sensitivity 7, 31, 32. Therefore, we transfected NCI-N87 cells to generate the MUC4-expressing GC cell line 87MUC4, and used it to establish animal models. Western blot studies demonstrated the increased MUC4 expression in 87MUC4 cells and tumor tissues (Figure 6A and Figure S5). The overexpression of MUC4 had little impact on the HER2 expression. The elevated MUC4 significantly inhibited the binding of ^99m^Tc-MIRC208 to 87MUC4 cells, but not ^99m^Tc-MIRC213. The binding of ^99m^Tc-MIRC208 in 87MUC4 cells was less than that in NCI-N87 cells (Figure 6B). The ^99m^Tc-MIRC208:^99m^Tc-MIRC213 ratio in 87MUC4 was 0.66 ± 0.01, and was significantly less than that in NCI-N87 (Figure 6C).

SPECT studies were performed to calculate the SUV_208_/SUV_213_ (2 h) ratio in the 87MUC4 tumor model. We found that the uptake of ^99m^Tc-MIRC208 in 87MUC4 tumors was significantly diminished, while there was little change in the uptake of ^99m^Tc-MIRC213 (Figure 6D-E). The SUV_208_/SUV_213_ (2 h) ratio (0.72 ± 0.02) in the 87MUC4 tumors was significantly lower than that in the NCI-N87 tumors (Figure 6F). A Traz treatment study was performed to compare the therapeutic effect of Traz on tumor growth rates of 87MUC4 and NCI-N87 (Figure 6G). The Traz therapy showed less inhibitory effect on the growth of 87MUC4 tumors than wild-type NCI-N87, indicating that increasing MUC4 expression could significantly decrease the Traz-sensitivity of NCI-N87. However, we found that Traz slightly inhibited the 87MUC4 tumor growth compared with saline group. This is maybe because the efficiency of overexpressing MUC4 receptor by lentivirus transfection is not 100%, resulting in a small number of wild and Traz-sensitive NCI-N87 cells in 87MUC4 tumors.

### The preliminary clinical study on ^99m^Tc-MIRC208

**Figure 7:**
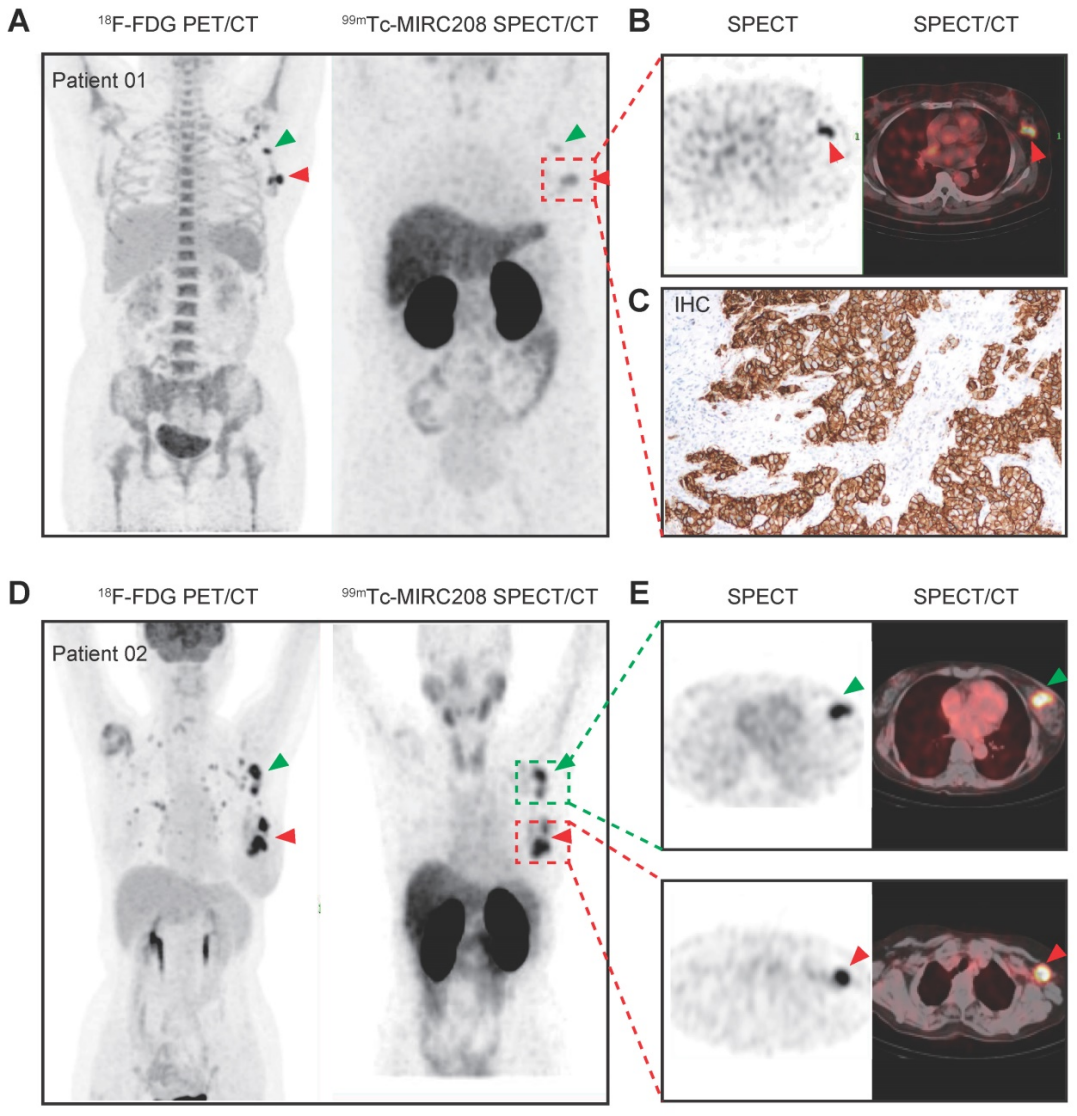
**^99m^Tc-MIRC208 SPECT/CT image and ^18^F-FDG PET/CT image in HER2-positive breast cancer patients.** Both patients were clinical stage III invasive left breast ductal carcinoma, and the HER2 IHC sore of the primary tumor is 3+. The patients didn't receive any treatment before imaging examination. **(A)** Representative maximum-intensity-projection (MIP) images of ^18^F-FDG PET/CT (left) and ^99m^Tc-MIRC208 SPECT (right) in patient 01. All of the primary and metastatic lesions exhibit high ^18^F-FDG metabolism. The primary lesion shows the highest uptake of ^99m^Tc-MIRC208. The metastatic lesion (red arrow) shows moderate uptake of ^99m^Tc-MIRC208, but is easily discernable from background. **(B)** The transaxial SPECT image and SPECT/CT fused images of the primary lesions in patient 01. **(C)** HER2 IHC staining of primary tumor of patient 01. **(D)** Representative MIP images of ^18^F-FDG PET/CT (left) and ^99m^Tc-MIRC208 SPECT (right) in patient 02. **(E)** The transaxial SPECT image and SPECT/CT fused images of the primary (indicated by red arrow) and metastatic (indicated by green arrow) lesions that exhibited high uptakes of ^99m^Tc-MIRC208 in D.

Prior to the clinical study, we performed safety assessment studies in mice. Blood sample testing and H&E staining of major tissues from the mice receiving a single high dose of radioactivity indicated that both radiotracers had favorable toxicity profiles (Figure S9-S10). Therefore, we carried out a preliminary clinical study of ^99m^Tc-MIRC208 in the HER2-positive BC patients (ClinicalTrials.gov identifier, NCT04591652). Two BC patients with HER2-positive tumors (HER2 IHC 3+) in the left breast have been enrolled up to date. In patient 01, ^99m^Tc-MIRC208 SPECT/CT clearly showed the HER2-positive primary tumor lesion and lymph node metastasis with a low background at 2 h p.i. (Figure 7A-B). The results of IHC staining showed HER2 is overexpressed in the primary tumor. In patient 02, the primary and large axillary lymph node metastatic lesions were clearly visualized by ^99m^Tc-MIRC208 SPECT/CT at 2 h p.i., but the pulmonary micro-metastatic lesions indicated by high ^18^F-FDG uptake didn't show significant ^99m^Tc-MIRC208 accumulation. Both patients had no serious adverse events or obvious vital sign changes within a week after ^99m^Tc-MIRC208 administration.

## Discussion

Considering the crucial role that epitope masking plays in Traz-resistance, it is imperative to identify potential Traz-responders 7, 8, 30, 33. However, standard clinical procedures for HER2 examination fail to satisfy this clinical need 13-16, 34. In this study, we proposed a dual radiotracer approach using two HER2-targeted ^99m^Tc radiotracers against different epitopes. We demonstrated that this approach is a powerful tool for noninvasive imaging of the HER2-positive tumors, selection of appropriate patients for Traz treatment, and prediction of Traz-resistance caused by epitope masking. The ^99m^Tc-labeling method used in this study is site-specific, and has many advantages, such as the single product and minimal impact on target affinity, which are perfect for the design of radiotracers for clinical applications 35-37. The final products could be established within only an hour with high radiopurity, HER2-binding affinity and solution stability (Figure 2 and Figure S3).

^99m^Tc-MIRC208 and ^99m^Tc-MIRC213 (Figure 3) recognize two different epitopes of HER2 receptor, and provide different diagnostic information concerning the molecular characteristics of tumors (Figure 4). ^99m^Tc-MIRC213 could be used to select HER2-positive patients and noninvasively quantify the tumor HER2 expression levels without the interference from MUC4. ^99m^Tc-MIRC208 was able to indirectly evaluate the HER2-accessibility of Traz. The SUV_208_/SUV_213_ (2 h) ratio can be a biomarker for selection of potential responders before therapy initiation. The patients with “^99m^Tc-MIRC213-positive” tumors tend to show a higher SUV_208_/SUV_213_ (2 h) ratio and would likely to benefit from Traz therapy. The patients with low SUV_208_/SUV_213_ (2 h) might be non-responders, and should consider other HER2-targeted therapies without Traz.

The radiotracers based on intact Traz, such as ^64^Cu-Traz or ^89^Zr-Traz, are the best molecular imaging tools for predicting HER2-accessibility of Traz 19, 38, 39. However, these antibody-based radiotracers are not suitable for widespread clinical applications due to their poor pharmacokinetic characteristics and long-term lag between administration and after examination 24, 25. In this study, ^99m^Tc-MIRC208, a Traz-competitor, was used as a substitute for the radiolabeled Traz. High quality SPECT images could be acquired with ^99m^Tc-MIRC208 as early as 0.5 h post injection, which helps the rapid design of an optimal therapeutic strategy and increases patient compliance with the examination. However, ^99m^Tc-MIRC208 alone cannot predict the HER2-accessibility of Traz, as it is less hindered by MUC4 than trastuzumab. It has been reported that MUC4 can sterically prevent intact trastuzumab from binding to nearly 80% of HER2 receptors on the JIMT-1 cell membrane 9. Because of the decreased molecular weight of VHH, only approximately 50% of HER2 is masked by MUC4 to ^99m^Tc-MIRC208 (Figure 5C), which may lead to confusing results when the tumor expresses an extremely high HER2 level. Thus, if we want to use ^99m^Tc-MIRC208 to determine whether MUC4 masks the Traz epitope, we must know the HER2 expression level in tumor in advance. That is the exact reason why we utilize the SUV_208_/SUV_213_ (2 h) ratios rather than the tumor uptake of ^99m^Tc-MIRC208 as the biomarker to predict the Traz-resistance before initiation of the Traz therapy.

Several other mechanisms also contribute to the Traz-resistance 2, 40. The Traz-mediated HER2 downregulation during treatment is an early prognostic indicator for therapeutic efficiency 41-43. Previously, we found that ^99m^Tc-HYNIC-H10F, a peptide-based radiotracer against subdomain II of HER2, was useful to predict the therapeutic response by noninvasively monitoring the decline in tumor HER2 expression after Traz therapy 44. ^99m^Tc-MIRC213 is a Traz non-competitor useful for noninvasive monitoring of tumor HER2 expression. Longitudinal monitoring of 7HER2-bearing mice undergoing Traz treatment revealed that the HER2 downregulation could also be detected noninvasively by ^99m^Tc-MIRC213 SPECT before any significant changes in the tumor size (Figure S8). ^99m^Tc-MIRC213 has higher HER2 binding affinity and better tumor uptake than ^99m^Tc-HYNIC-H10F. Therefore, ^99m^Tc-MIRC213 has better sensitivity and accuracy in evaluation of the changes in HER2 expression. ^99m^Tc-MIRC213 could also be utilized to predict the treatment response of patients who have been defined as responders by our imaging approach and are being treated by Traz, which can further promote the design of personalized therapeutic approaches and improve patient management.

^99m^Tc-MIRC208 SPECT/CT studies were carried out in two HER2-positive breast cancer patients. At 2 h p.i., the HER2-positive primary tumor lesion and lymph node metastasis were clearly visualized with very low background. However, several micro-metastatic lesions indicated by ^18^F-FDG PET were not visualized by ^99m^Tc-MIRC208 SPECT, probably due to low HER2 expression in these lesions or poor sensitivity of SPECT as compared with PET. Since the pathological data of metastatic lesions are not available at the moment, this explanation remains speculative. It should be investigated in the future. High radioactivity accumulation was also observed in kidneys. No adverse events or abnormal vital signs were observed after injection, confirming that ^99m^Tc-MIRC208 is safe and well-tolerated in patients.

Despite the outstanding results, there are several important limitations in this study. JIMT-1 is the only natural biological model we used to test the predictive validity of our dual radiotracer approach. More preclinical models that naturally co-express MUC4 and HER2 receptors should be enrolled in the future. Moreover, the Traz-resistance caused by other epitope-masking receptors, such as CD44/hyaluronan, has yet to be investigated by our imaging approach. A large-scale clinical study of ^99m^Tc-MIRC208 SPECT and the clinical translation of ^99m^Tc-MIRC213 SPECT should be conducted in the future. The correlation between the SUV_208_/SUV_213_ (2 h) ratio and the Traz-sensitivity in cancer patients remains to be explored in future clinical studies.

## Conclusion

In summary, we present a proof-of-concept for the dual radiotracer approach to predict the Traz-resistance caused by epitope masking. We provide a powerful tool for assessment of the expression levels of both membrane and Traz-bound HER2. This tool could effectively stratify patients for Traz-therapy before treatment by rapidly screening HER2-positive patients and predicting their Traz-responsiveness, thus ultimately guiding personalized therapy and reducing toxicity and cost of unnecessary therapies that don't contribute to patient benefit.

## Methods and Materials

All animal studies were performed according to the protocols approved by the Institutional Animal Care and Use Committee at Peking University. Detailed information on cell cultures, animal models, HER2-trageted VHHs generation, radiotracers preparation, SPECT imaging and tumor treatment is provided in Supplementary Information.

### First-in human study with ^99m^Tc-MIRC208

The Institutional Review Board of Peking University Cancer Hospital & Institute approved this study (#2019KT114, NCT04591652), and all subjects signed a written informed consent. To data, two patients with clinical stage III invasive left breast ductal carcinoma were enrolled in this study. Patient 01 and patient 02 are women aged 55 and 65, respectively. The HER2 overexpression of primary tumors in both patients had been confirmed by IHC using the commercial test kit (IHC 3+). Both patients were enrolled without any prior treatment.

^99m^Tc-MIRC208 SPECT and ^18^F-FDG PET/CT were acquired within 3 days for comparison. For ^18^F-FDG PET/CT, patient fasted for at least 6 h before intravenous injection of ^18^F-FDG at a dosage of 5.6 MBq/kg of body weight. ^18^F-FDG PET/CT scan was performed 1 h post injection with a PET/CT scanner (Philips Medical Systems). ^99m^Tc-MIRC208 was freshly prepared in the morning of imaging day and the radiochemical purity was proved with ITLC method to be greater than 95% before use. ^99m^Tc-MIRC208 was given by intravenous injection and the injected activity was 14.2 MBq/kg. The total injection doses of patient 01 and patient 02 were 880.4 MBq and 823.6 MBq, respectively. A chest to abdomen SPECT/CT scans was performed at 2 h after ^99m^Tc-MIRC208 injection with a SPECT/CT scanner (Symbia T16; Siemens). A siemens workstation (MultiModality Workplace) was used for data processing. The images were evaluated and quantified by two experienced nuclear medicine physicians.

### Statistical analysis

Detailed information on sample numbers and statistical tests used are described in the figure legends. Calculation was performed using GraphPad Prizm 8.0 software. The differences between two groups were tested with student's paired *t test*. Multiple comparison was done with two-way analysis of variance (ANOVA) followed by a Bonferroni* post hoc* test. *P* value of 0.05 or lower was considered statistically significant. n.s. indicates not significance.

## Supplementary Material

Supplementary materials and methods, figures.
