# Supplementary material for: HER2-targeted dual radiotracer approach with clinical potential for noninvasive imaging of trastuzumab-resistance caused by epitope masking
Source: Theranostics. 2022 Jul 18;12(12):5551–63. doi: 10.7150/thno.74154 (PMC9330517; doi:10.7150/thno.74154)
Supplement: Supplementary file 1 — Supplementary materials and methods, figures. [file thnov12p5551s1.pdf]

Supplementary information for

HER2-Targeted dual radiotracer approach with clinical potential for noninvasive imaging of trastuzumab-resistance caused by epitope masking

Liqiang Li[a,b], Tianyu Liu[a], Linqing Shi[a], Xin Zhang[a], Xiaoyi Guo[b], Biao Hu[a], Meinan Yao[a], Hua Zhu[b], Zhi Yang[b], Bing Jia[a,c,1], Fan Wang[a,d,e]

[a]Medical Isotopes Research Center and Department of Radiation Medicine, School of Basic Medical Sciences, Peking University, Beijing, 100191, China.

[b]Key Laboratory of Carcinogenesis and Translational Research (Ministry of Education/Beijing), Department of Nuclear Medicine, Peking University Cancer Hospital & Institute, Beijing 100142, China.

[c]Department of Integration of Chinese and Western Medicine, School of Basic Medical Sciences, Peking University, Beijing, 100191, China.

[d]Key Laboratory of Protein and Peptide Pharmaceuticals, CAS Center for Excellence in Biomacromolecules, Institute of Biophysics, Chinese Academy of Sciences, Beijing 100101, China.

[e]Bioland Laboratory (Guangzhou Regenerative Medicine and Health Guangdong Laboratory), Guangzhou 510005, China.

[1]To whom correspondence may be addressed. Email: jiabing@bjmu.edu.cn

Author Contributions: L.Q.L. and B.J. designed the experiments; L.Q.L., T.Y.L., L.Q.S., B.H., X.Z., and M.N.Y. performed the preclinical studies; X.Y.G., H.Z., and Z.Y. performed the polity clinical

studies; L.Q.L., B.J. and F.W. analyzed the data; L.Q.L., B.J., and F.W. drafted and edited; J.B. and F.W. supervised the research.

This PDF file includes:

Methods for supplementary figures

Figures S1 to S10

Methods for supplementary figures

Chemicals and biochemicals

Recombinant human HER2 ECD protein (HER2-H5225) was purchased from ACRO Biosystems. Human EGFR ECD (10001-H08H-B), HER3 ECD (10201-HCCH), HER4 ECD (10363-H08B) and mouse HER2 ECD (50714-M08H) proteins were purchased from Sino Biological. Anti-human HER2 antibody (2165S) was from Cell Signaling Technology, and anti-human MUC4 antibody (354900) was purchased from ThermoFisher Scientific. The pCDH-hHER2-blasticidin lentivirus was kindly provided by Pro. Pengyuan Yang in Institute of Biophysics, Chinese Academy of Sciences, and pLebt-CMV-hMUC4-blasticidin lentivirus (variant 4) (plv20101101) was purchased from Vigene Biosciences Company. GGGGEC oligopeptide (682554) was from GL Biochem. Cell counting kit-8 (CCK8, CK04) was from Dojindo Molecular Technologies. Trastuzumab (Traz) was purchased from Roche. Matrigel was from BD Biosciences. BSA (CA003) was purchased from Macgene. GH kit was from Beijing ShiHong Company. Anti-β-actin antibody (sc-47778) was from Santa Cruz. HRP goat anti-mouse antibody (AS003) and HRP goat anti-rabbit antibody (AS014) were purchased from ABclonal. X-ray films (6535876) were from Eastman Kodak Company. Blasticidin (B9300) was from Solarbio. High affinity Ni-NTA resin was from GenScript. RIPA buffer (89900) was from Thermo Scientific. Protease inhibitor cocktail (P8849-1ML) was from Sigma. BCA protein assay kit (MPK002) was from Macgene.

Cell cultures and animal models

MDA-MB-453 (M-453) and MCF-7 were purchased from the American Type Culture Collection (Manassas, VA). JIMT-1 was form AddexBio Technologies Company (San Diego, American). NCI-N87 was kindly provided by Pro. Zhi Yang in Beijing Cancer Hospital. 7HER2 was acquired by transfecting MCF-7 with pCDH-hHER2-blasticidin lentivirus. MUC4-overexpressed 87MUC4 was

acquired by infecting NCI-N87 with plebt-CMV-hMUC4-blasticidin lentivirus. 7HER2, MCF-7 and JIMT-1 were cultured in high-glucose DMEM. NCI-N87, 87MUC4 and M-453 were cultured in RPMI-1640 medium. All cells were mycoplasma free and cultured with suitable medium containing 10% fetal bovine serum in humidified incubator with 5% CO_2 at 37°C. The expression levels of MUC4 and HER2 in cell cultures were verified by immunoblotting studies with anti-human MUC4 antibody and anti-human HER2 antibody, respectively.

NOD/SCID and BALB/c nude mice were purchased from Beijing Vital River Laboratory Animal Technology (Beijing, China). All animal experiments were performed in accordance with the guidelines of the Peking University Animal Care and Use Committee. NCI-N87 and 87MUC4 tumor models were established by subcutaneously injecting female BALB/c mice with 1×10^7 and 1.5×10^7 cells suspended in 1:1 v/v mixture of medium with Matrigel, respectively. To obtain 7HER2 and MCF-7 tumor models, 5×10^6 in 100 μL PBS 7HER2 or MCF-7 cells were inoculated subcutaneously into the right front flanks of female NOD/SCID mice. Because MCF-7 and 7HER2 tumor growth is estrogen dependent, 0.72 mg 17β-estradiol pellet for 60-day release was implanted subcutaneously in each mouse 2 days before cell inoculation. The animals were used for *in vivo* SPECT/CT and biodistribution studies when the tumor size reached 100-150 mm^3 (1-2 weeks after inoculation). The Traz treatment studies were initiated at 4 d post inoculation.

Generation of the HER2-targeted VHHs

To acquire the novel HER2-targeted VHHs, we immunized a health alpaca with recombination protein of HER2 ECD using a standard procedure. Briefly, an alpaca was immunized in a weekly regimen four times with HER2 ECD protein (1 mg for the first time and 0.5 mg for the other three times. VHH libraries were constructed from peripheral blood lymphocytes as described elsewhere [1].

VHH selections were done using a total of two rounds of panning performed on solid-phase immobilized antigen. Sequence analysis of ELISA-positive clones yielded 42 candidates. The sequence homology analysis of VHHs was carried out using online Basic Local Alignment Search Tool (BLAST) of NCBI website. The BLAST result was presented as phylogenetic trees and modified by Evolview v3 webserver [2].

To evaluate the HER2 affinities of 10 representative VHH, we performed SPR study with HER2 ECD protein. The SPR measurements were performed on a PlexArray HT (version 3, Plexera, American) instrument. Traz and a VHH against other receptor were chosen as the positive and negative controls, respectively. VHHs and Trazwere coupled on a 3D Dextran chip as stationary phase. Recombinant human HER2 ECD protein was used as mobile phase and flown at 2 μL/s in PBS. The chip was regenerated using 10 mM Gly-HCL (pH 2.0). For determination of binding kinetics, mobile phase concentration was set to 30 nM, 60 nM, 120 nM, 240 nM and 480 nM. Binding was allowed for 300 s and dissociation for 300s. The curves were fitted by PlexeraDE software (Plexera, American) and the association rate constants (Ka), dissociation rate constants (Kd), and equilibrium dissociation constants (KD, a measurement of affinity) were determined by 1:1 antigen:analyte binding model.

Preparation ^{99m}Tc Radiotracers

We firstly prepared the radiolabeling precursors by c-terminal site-specifically tagging VHHs with G_4EC oligopeptides using Sortase A. Briefly, purified VHH (0.32 mM) was incubated with Sortase A (4 μM) and G_4EC oligopeptide (10 mM) in Sortase buffer containing 50 mM Tris-HCL, pH 7.5, 150 mM NaCl, 10 mM $CaCl_2$ for 12 h at 4 °C. To remove excess Sortase A and unreacted VHH, the reaction was incubated with Ni-NTA beads with agitation for 30 min at 4 °C followed by centrifugation. The VHH-G_4EC was purified by size-exclusion chromatography in 0.1 M PBS solution and stored at

-80 °C. SDS-PAGE studies were used to verify the purities of three VHH-G_4EC and LC-MS analysis confirmed successfully tagging.

Then, the radiolabeling of VHH-G_4EC with ^{99m}Tc was performed following an establishment procedure in which ^{99m}Tc-GH was used as intermediate [3]. ^{99m}Tc-GH was acquired according to the manufacture' protocol. 150 μg VHH-G_4EC in 0.1 M PBS (100 μL) was mixed with 100 μL 0.5 M succinic acid buffer (pH=4.7) and 300 μL ^{99m}Tc-GH (typically, 370 MBq-555 MBq). The mixture was thoroughly vortexed and incubated at 37 °C for 0.5 h. After that, the mixture was loaded onto a NAP5 size-exclusion cartridge and eluted with sterile saline yielding > 70% of ^{99m}Tc-VHH (decay-corrected radiochemical yield). The radiochemical purity of tracer was determined by radio-HPLC with superdex 75 column.

Three ^{99m}Tc-VHHs were incubated in saline for 0, 2, 4 and 6 h at 25°C to test the *in vitro* stability. The samples were analyzed by radio-HPLC. For the *in vivo* stability assays, female BALB/c mice were injected with ^{99m}Tc-VHHs at a dose of 74MBq in 0.1 mL PBS via tail vein. At 6 h time point, the blood samples were collected via retro-orbital blood draws. The blood samples were centrifuged at 8000 rpm for 10 min. The supernatants were collected, filtered through a 0.22 μ filter, and then analyzed by radio-HPLC.

In vitro evaluations

To test the specificity of three ^{99m}Tc-VHHs to HER2 receptor, we firstly conducted the protein binding assay with the human receptors of HER family and murine HER2 receptor. Briefly, 0.2 μg HER family protein (human EGFR, human HER2, human HER2, human HER4 and mouse HER2 ECD protein) was immobilized on immunosorbent plates and blocked with 2% BSA solution. HER family protein was incubated with ^{99m}Tc-MIRC208, ^{99m}Tc-MIRC213 and ^{99m}Tc-MIRC220 (11 kBq/per well) at

4 °C for 60 min. For blocking studies, all binding assays were performed with quadruplicate wells. Protein binding was expressed as a percentage of total added radioactive dose after decay correction (%AD).

Secondly, we performed the cell binding assays with multiply types of BC and GC cell lines. The NCI-N87, 87MUC4, 7HER2, M-453, JIMT-1 and MCF-7 cells were seeded into 24-well plates at a density of 5×10^5 per well overnight and washed three times with ice-cold PBST prior to addition of ^{99m}Tc-VHHs. Cell uptake study was measured by incubating cells with ^{99m}Tc-MIRC208, ^{99m}Tc-MIRC213 or ^{99m}Tc-MIRC220 (11 kBq/ 1.0 per well) at 4 °C for 60 min. For the blocking studies, radiotracers were challenged with 1000-fold molar excess of homologue VHH or Traz. After incubation, cell was solubilized in 2 M NaOH, and the total lysis was collected for the radioactivity measurement with a γ-counter. All cell uptake studies were performed with quadruplicate wells. Cell uptakes were expressed as a percentage of total added radioactive dose after decay correction.

In vivo evaluations

For SPECT/CT imaging, tumor-bearing mice received an intravenous injection of approximately 20 MBq (540 μCi in 100 μL PBS) ^{99m}Tc-VHH and underwent the SPECT/CT scan (Mediso Inc., Hungary) at indicated time points. During the SPECT/CT scan, mice were anesthetized by inhalation of 2% isoflurane in O_2. The pinhole SPECT images (peak: 140 keV, 20% width; frame time: 20 s) were acquired for 18.5 mins and subsequently CT images were acquired (50 kVp, 0.67 mA, rotation 210°, exposure time: 300 ms).

For biodistribution studies, tumor-bearing mice were injected with about 0.222 MBq (6 μCi in 100 μL) of ^{99m}Tc-VHHs through the tail vein, and then sacrificed and dissected at indicated time points. Tumors and major organs were collected and weighted. The radioactivity in the wet whole tissue was

measured with a γ-counter and the results were expressed as %ID/g.

Firstly, we assessed the *in vivo* properties of three ^{99m}Tc-VHHs by biodistribution studies in 7HER2 tumor models. The mice bearing 7HER2 tumors were intravenously injected with thee ^{99m}Tc-VHHs and then sacrificed and dissected at 1 h, 2 h, 4 h, 6 h after injection.

Secondly, to explore the abilities of thee ^{99m}Tc-VHHs to image HER2-positive tumors, we performed the SPECT/CT imaging studies in the 7HER2 tumor models injected with ^{99m}Tc-MIRC208, ^{99m}Tc-MIRC213 or ^{99m}Tc-MIRC220 at 0.5 h, 1 h, 2h and 4 h post injection.

Thirdly, we preformed the *in vivo* blocking studies in 7HER2 tumor models to test the HER2 specificities of ^{99m}Tc-MIRC208 and ^{99m}Tc-MIRC213. For blocking SPECT/CT imaging, ^{99m}Tc-VHH was co-injected with excess homologue VHH (400 μg in 100 μL PBS) into animals, and imaging studies were performed at 2 h post injection. For blocking biodistribution studies, ^{99m}Tc-VHH was co-injected with excess homologue VHH (400 μg in 100 μL PBS) into animals, and animals were sacrificeed at 2 h post injection.

Fourthly, we performed the *in vivo* studies in HER2-low MCF-7 tumor models to further explore the specificities of ^{99m}Tc-MIRC208 and ^{99m}Tc-MIRC213 to HER2 receptor. The SPECT/CT imaging and biodistribution studies were performed in MCF-7 tumor models at 2 h post administration of ^{99m}Tc-VHHs.

Investigation of binding epitopes of ^{99m}Tc-MIRC208 and ^{99m}Tc-MIRC213

To investigate the epitopes of ^{99m}Tc-MIRC208 and ^{99m}Tc-MIRC213 on HER2 receptor, we firstly preformed *in vitro* blocking studies with HER2 ECD protein and HER2-high 7HER2 cells in the presence of excess Traz or homologue VHH. ^{99m}Tc-VHHs were challenged with 1000-fold molar excess of homologue VHH or Traz. The experimental operations are the same as those described

above.

Afterwards, we conducted the *in vivo* blocking imaging studies in 7HER2 tumor models. For Traz blocking studies, excess Traz (2 mg in 100 µl PBS) was intravenously injected 48 h prior to the administration of radiotracers. For homologue VHH blocking studies, homologue VHH (400 µg in 100 µL PBS) was co-injected with ^{99m}Tc-VHH. The SPECT/CT imaging studies were performed at 2 h post injection. SPECT/CT fusion images were obtained, and ROIs were measured using Fusion software (Mediso Inc., Hungary). The values of tumors and other normal tissues uptake were expressed as the %ID/cc.

The dual radiotracer strategy

For the combinational SPECT/CT imaging approach, the ^{99m}Tc-MIRC213 SPECT/CT imaging at 2 h post injection was firstly conducted in the tumor bearing mouse, and 24 h after, ^{99m}Tc-MIRC208 SPECT/CT was performed in the identical mouse at 2 h post injection. We performed the imaging approach in the mice bearing 7HER2, NCI-N87, JIMT-1 and 87MUC4 tumors.

To further verify the difference in the uptakes of two ^{99m}Tc-VHHs in the same types of tumors, we performed biodistribution studies at 2 h post injection in 7HER2, NCI-N87, JIMT-1 and 87MUC4 tumor models.

In vitro Traz-treatment assays

To test the sensitivities of 7HER2, NCI-N87, JIMT-1 and MCF-7 to Traz treatment, we firstly conducted the *in vitro* therapeutic studies. Tumor cells pre-seeded in 96-well dish (2000 cells per well) were treated with the indicated concentrations of trastuzumab, and 72 h later, the relative numbers of surviving cells were measured with CCK-8 according to the manufacturers' instructions.

In vivo therapeutic assays

The *in vivo* Traz treatment was initiated at 4 d post tumor inoculation. Mice were randomly divided into control group and treatment group based on the tumor volumes (7 mice per group). Mice in treatment group were given Traz (150 μg per mouse) intraperitoneally weekly for 3 weeks, and mice in control group were given corresponding vehicle (sterilized saline). Tumor growth was measured routinely with a Vernier caliper and the volume was defined as 1/2 × length × width^2.

Toxicity of ^{99m}Tc-VHHs

Female and male BALB/c mice were fed under normal conditions and randomly divided into two groups (four mice per group). Four male mice and four female mice were injected with an overdose (100 mCi/kg) of ^{99m}Tc-MIRC208 or ^{99m}Tc-MIRC213 as experimental group. Four male mice and four female mice were injected with 0.1 ml sterile saline as control group. The entire injection procedure should be completed in 5 seconds. The mice of two groups were observed. The blood biochemical parameters of two groups were acquired at -1, 0, 1, 3, 6, 10 and 15 d p.i.. The blood samples for the toxicity study of ^{99m}Tc-VHH were collected via retro-orbital blood draws. At 16 d p.i., all the mice were sacrificed and the status of the major organs were determined by HE staining.

Monitoring tumor response to Traz by ^{99m}Tc-MIRC213 SPECT/CT imaging

Animal studies were conducted to monitor tumor response and Traz-mediated HER2 downregulations in mice bearing dual MCF-7 (left) and 7HER2 (right) tumors. The Traz therapy plan was same as previously described and treatment was initiated on 4 d post inoculation (scheme shown in Fig. S7A). The longitudinal ^{99m}Tc-MIRC213 SPECT studies were performed at 4 d, 11 d and 18 d post inoculation (3 mice of each group). Tumor uptakes (%ID/cc) from SPECT images were measured. The differences in tumor uptakes between control and treatment groups were calculated on imaging days.

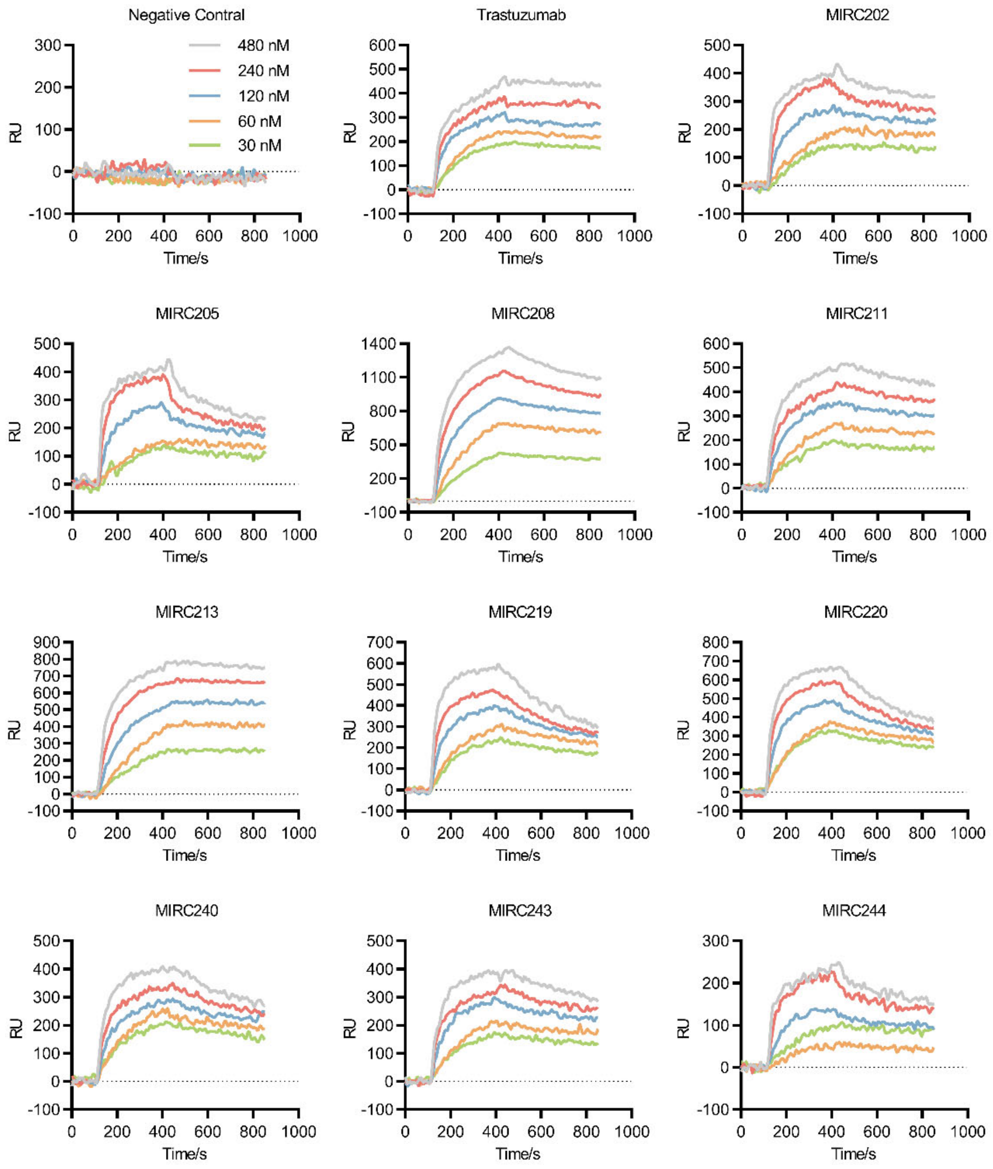

Supplementary Figure S1, related to Figure 1D.

The graphs of SPRi measurement that reveals the HER2 affinities of 10 VHHs. A VHH targeting other receptor and trastuzumab are enrolled into this study as negative and positive controls, respectively.

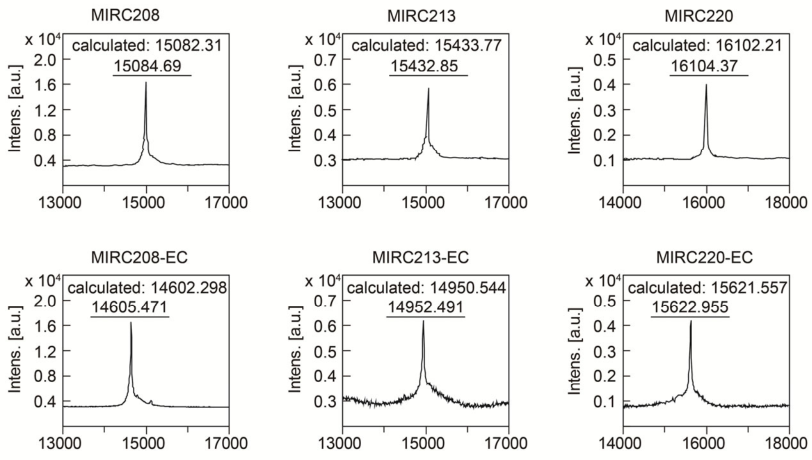

Supplementary Figure S2

MALDI-TOF mass spectrums of primary VHHs and VHH-ECs.

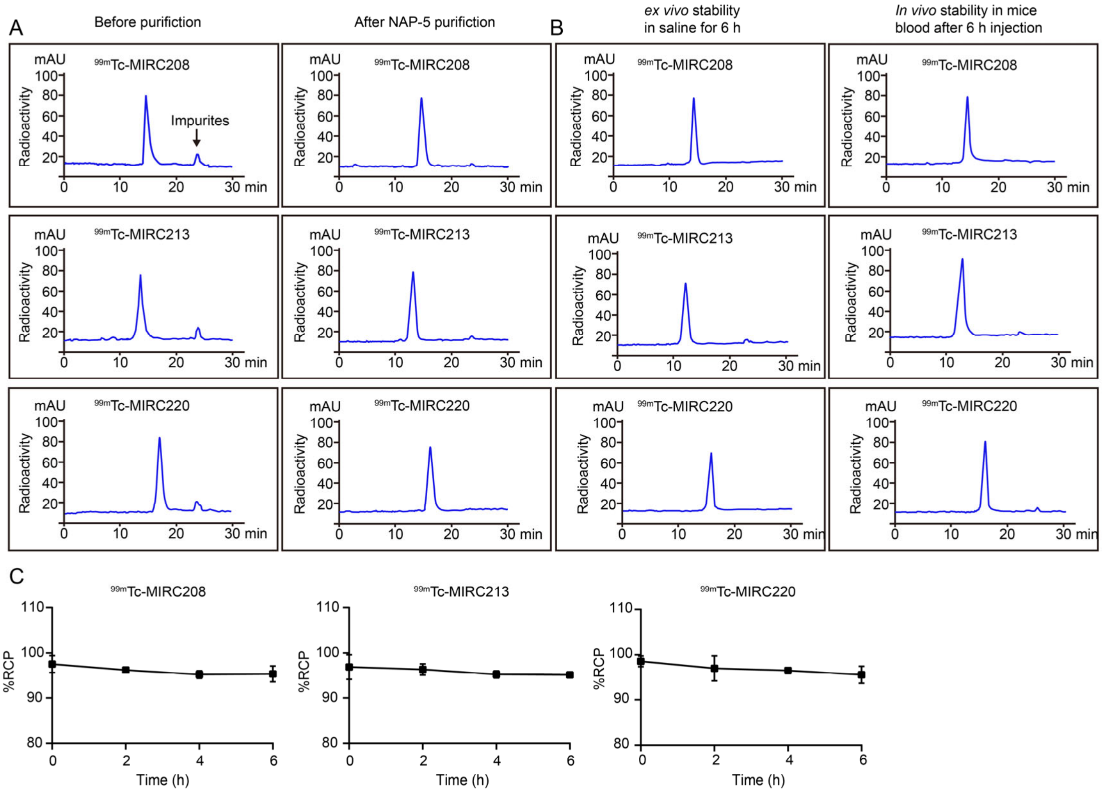

Supplementary Figure S3

(A) The typical radio-HPLC results of the radiopurities of ^{99m}Tc-VHHs before and after purification.

(B) The typical radio-HPLC results of *in vitro* solution stability in saline and *in vivo* stability in mice blood. (C) The quantitative data of ^{99m}Tc-VHHs in saline for 0 h, 2 h, 4 h and 6 h.

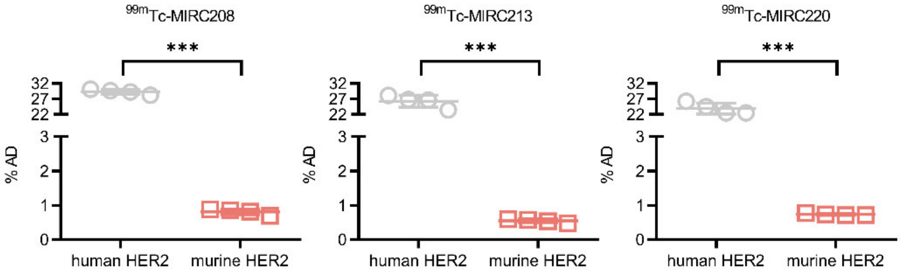

Supplementary Figure S4

The binding affinities of three ^{99m}Tc-VHHs to human and murine HER2 ECD proteins.

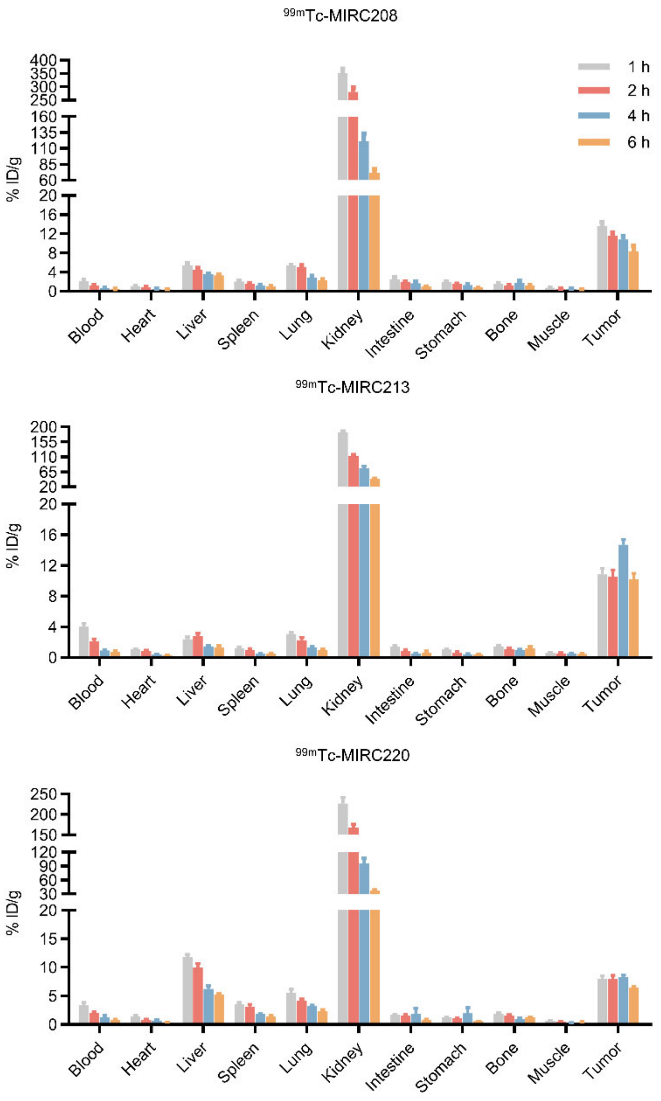

Supplementary Figure S5, related to Figure 3A and B

Biodistribution studies of three ^{99m}Tc-VHHs in 7HER2 tumor models at 1 h, 2 h, 4 h and 6 h post injection.

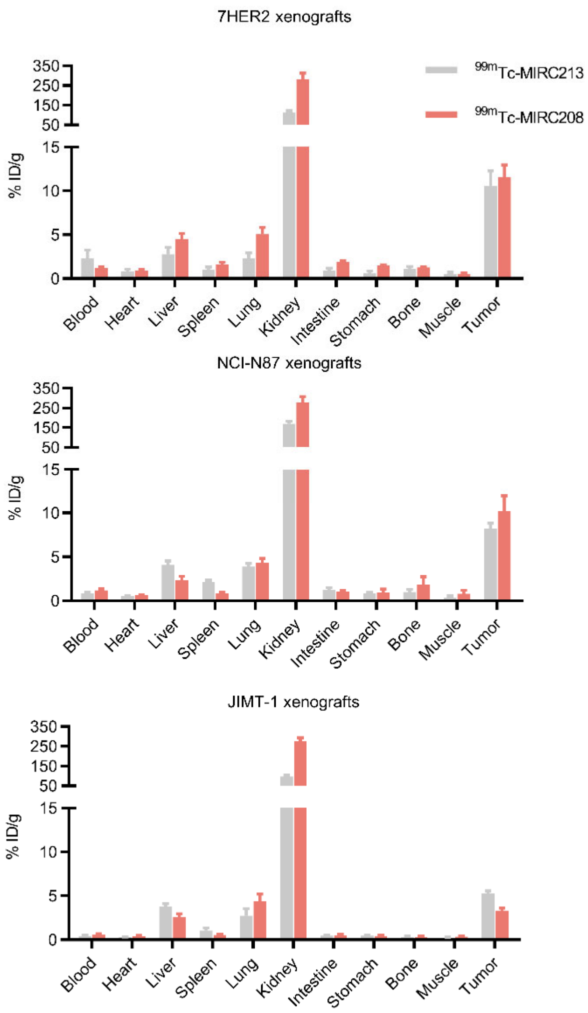

Supplementary Figure S6, related to Figure 5G

Biodistribution studies of ^{99m}Tc-MIRC208 and ^{99m}Tc-MIRC213 in 7HER2, NCI-N87 and JIMT-1 tumor models at 2 h post injection.

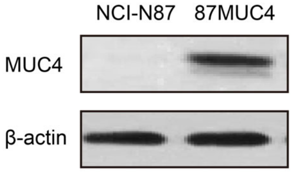

Supplementary Figure S7

The results of MUC4 immunoblotting of NCI-N87 and 87MUC4 tumor tissues.

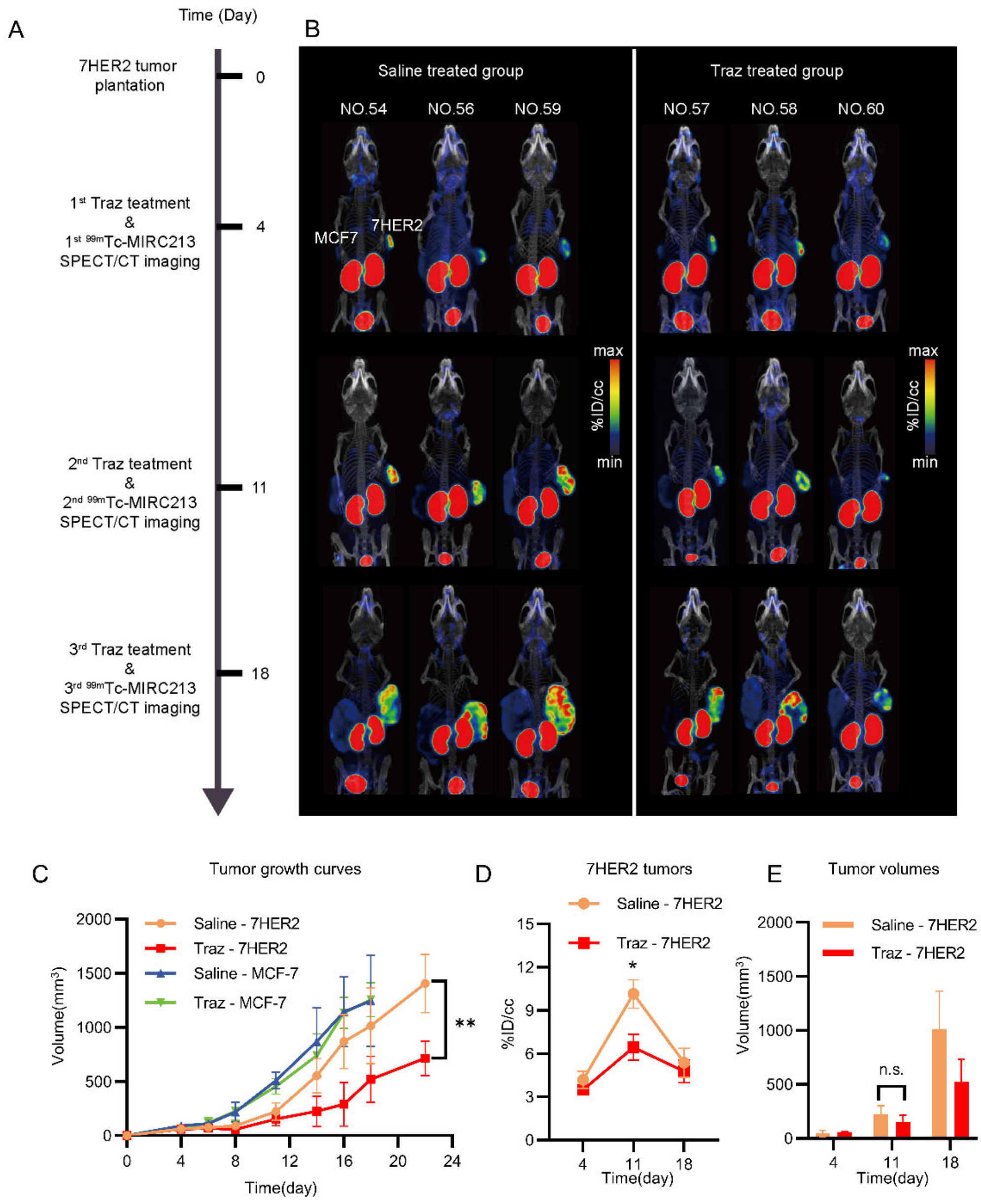

Supplementary Figure S8 Monitoring Traz-mediated HER2 downregulation in 7HER2 xenografts during treatment by ^{99m}Tc-MIRC213 SPECT.

(A) Schematic diagram of dual 7HER2 (right) and MCF7(left) tumor-bearing mice treated with Traz and used in imaging studies with ^{99m}Tc-MIRC213. Traz treatment (200 μg per mouse) and ^{99m}Tc-MIRC213 SPECT (20 MBq per mouse and images are acquired at 2h post injection) are performed in xenografts at 4 d, 11 d and 18 d after tumor implantation. (B) A series of ^{99m}Tc-MIRC213 SPECT/CT images in Traz-treated xenografts (n=3). (C) Tumor growth curves of 7HER2 and MCF7

treated by Traz(n=7). Tumor volumes are expressed as mean ± SD (n = 7). **$p < 0.01$, two-way analysis ANOVA followed by a Bonferroni *post hoc* test. (D) Quantitative analyses of ^{99m}Tc-MIRC213 accumulations in 7HER2 during treatment. error bars represent SD. *$p < 0.05$, student's paired *t test*. (tumor uptakes at 11 d were compared between Traz and saline groups) (E) Tumor volumes of 7HER2 treated with Traz or saline at 4 d, 11 d and 18 d post implantation. error bars represent SD. n.s., no significance, $p > 0.05$, student's paired *t test* (tumor volumes at 11 d were compared between Traz and saline groups) .

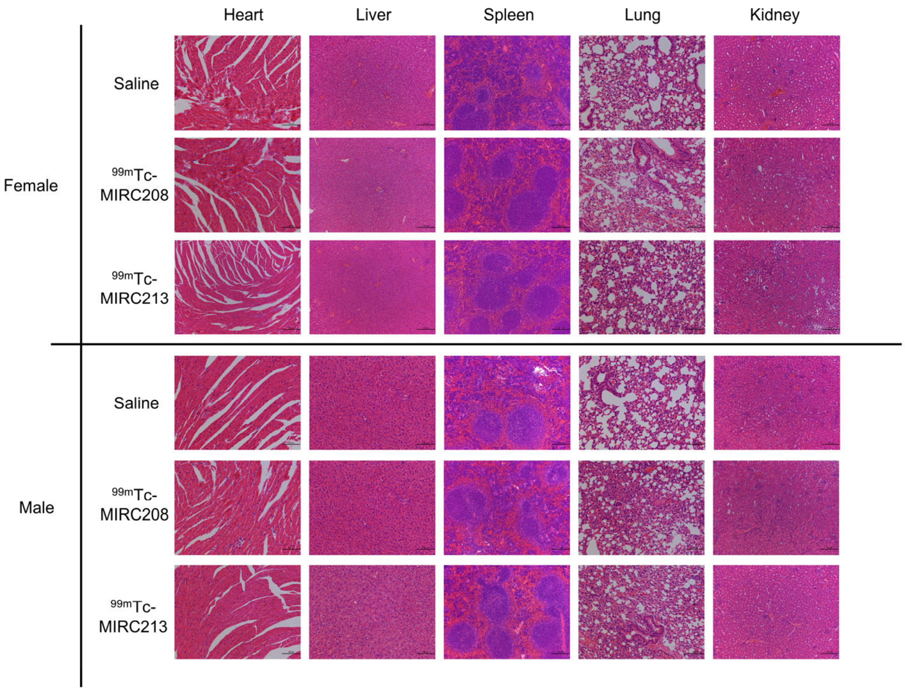

Supplementary Figure S9

HE staining of major tissue samples from mice receiving saline and single high drug does. Male and female (B) mice in the treating group are administrated with 100 mCi/kg ^{99m}Tc-MIRC208 or ^{99m}Tc-MIRC208 (normal dose: 2 mCi/kg calculated with species specificity).

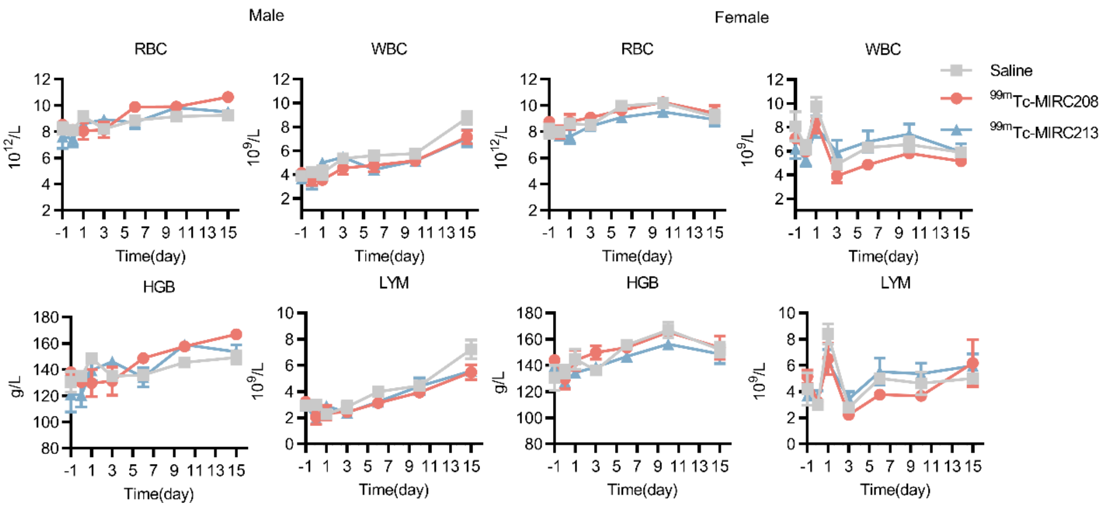

Supplementary Figure S10

Analysis of blood samples of mice receiving saline or single high doses radioactivity. Representative blood parameters are shown in this figure.

Male (left) and female (right) mice in the radioactivity-treated group are administrated with 100 mCi/kg ^{99m}Tc-MIRC208 or ^{99m}Tc-MIRC208 (normal dose: 2 mCi/kg calculated with species specificity). RBC: red blood cell; WBC: white blood cell; HGB: hemoglobin; LYM: lymphocyte. There are no changes in representative blood parameters between saline and radioactive treating groups.

Reference

1. Pardon E, Laeremans T, Triest S, Rasmussen SG, Wohlkonig A, Ruf A, et al. A general protocol for the generation of Nanobodies for structural biology. Nat Protoc. 2014; 9: 674-93.
2. Subramanian B, Gao S, Lercher MJ, Hu S, Chen W-H. Evolview v3: a webserver for visualization, annotation, and management of phylogenetic trees. Nucleic Acids Res. 2019; 47: W270-W5.
3. Oroujeni M, Andersson KG, Steinhardt X, Altai M, Orlova A, Mitran B, et al. Influence of composition of cysteine-containing peptide-based chelators on biodistribution of (99m)Tc-labeled anti-EGFR affibody molecules. Amino Acids. 2018; 50: 981-94.
